# Identification and validation of an immune signature associated with EMT and metabolic reprogramming for predicting prognosis and drug response in bladder cancer

**DOI:** 10.3389/fimmu.2022.954616

**Published:** 2022-07-25

**Authors:** Zhao Zhang, Yongbo Yu, Peng Li, Meilan Wang, Wei Jiao, Ye Liang, Haitao Niu

**Affiliations:** ^1^ Department of Urology, The Affiliated Hospital of Qingdao University, Qingdao, China; ^2^ Key Laboratory, Department of Urology and Andrology, The Affiliated Hospital of Qingdao University, Qingdao, China; ^3^ Nursing department, Shandong Institute of Petroleum and Chemical Technology, Dongying, China

**Keywords:** epithelial-mesenchymal transition, metabolic reprogramming, prognosis, gene signature, bladder cancer

## Abstract

**Background:**

Epithelial-mesenchymal transition (EMT), one leading reason of the dismal prognosis of bladder cancer (BLCA), is closely associated with tumor invasion and metastasis. We aimed to develop a novel immune−related gene signature based on different EMT and metabolic status to predict the prognosis of BLCA.

**Methods:**

Gene expression and clinical data were obtained from TCGA and GEO databases. Patients were clustered based on EMT and metabolism scores calculated by ssGSEA. The immune-related differentially expressed genes (DEGs) between the two clusters with the most obvious differences were used to construct the signature by LASSO and Cox analysis. Time-dependent receiver operating characteristic (ROC) curves and Kaplan–Meier curves were utilized to evaluate the gene signature in training and validation cohorts. Finally, the function of the signature genes AHNAK and NFATC1 in BLCA cell lines were explored by cytological experiments.

**Results:**

Based on the results of ssGSEA, TCGA patients were divided into three clusters, among which cluster 1 and cluster 3 had completely opposite EMT and metabolic status. Patients in cluster 3 had a significantly worse clinical prognosis than cluster 1. Immune-related DEGs were selected between the two clusters to construct the predictive signature based on 14 genes. High-risk patients had poorer prognosis, lower proportions of CD8^+^ T cells, higher EMT and carbohydrate metabolism, and less sensitivity to chemotherapy and immunotherapy. Overexpression of AHNAK or NFATC1 promoted the proliferation, migration and invasion of T24 and UMUC3 cells. Silencing ANHAK or NFATC1 could effectively inhibit EMT and metabolism in T24 and UMUC3 cells.

**Conclusion:**

The established immune signature may act as a promising model for generating accurate prognosis for patients and predicting their EMT and metabolic status, thus guiding the treatment of BLCA patients.

## Introduction

Bladder cancer (BLCA) is one of the most common malignant tumors of urinary system, and its incidence rate ranks tenth among all cancers and is gradually increasing, especially in the aging population. It is more common in men than in women, with morbidity and mortality rates about four times higher in men than in women (1). According to the depth of tumor infiltration, bladder cancer can be divided into non-muscle invasive bladder cancer (NMIBC) and muscle invasive bladder cancer (MIBC). NMIBC accounts for about 75% and MIBC accounts for about 25%. Despite the continuous development of surgery combined with chemoradiotherapy and immunotherapy techniques, the recurrence and metastasis rates of bladder cancer are still high, resulting in a poor prognosis (2). An important reason for this phenomenon is that bladder cancer is highly heterogeneous and varies greatly among individual patients, who can exhibit multidrug resistance (3). Current conventional methods still have difficulty in accurately predicting the prognosis of patients with BLCA. Therefore, with the increasing development of genomics, there is an urgent need to develop more effective and reliable prognostic biomarkers to distinguish different subgroups of patients and to enable optimal and personalized treatment.

Invasion and metastasis are unique features of BLCA that affects the survival and prognosis of patients and is also an important reason why surgery cannot completely remove the tumor lesion. An important process that precedes the development of tumor metastasis is epithelial-mesenchymal transition (EMT), caused by alterations in the molecular pathways of the tumor resulting from genetic and epigenetic changes (4). EMT is an embryonic phenotypic plasticity program that confers aggressiveness, dissemination, and chemo/immunotherapy resistance in cancer. Accompanied with the loss of apicobasal polarity and increased migratory capacity, EMT can make fixed and immobile urothelial cells undergo complex reprogramming, and make some urothelial cancer cells obtain mesenchymal characteristics with self-renewal capacity, so as to escape immune surveillance and penetrate into the surrounding basement membrane (5, 6). In contrast to epithelial cells, mesenchymal carcinoma cells have distinct metabolic demands that drive metabolic reprogramming throughout the tumor microenvironment. During EMT occurrence, cancer cells fine-tune their multiple metabolic circuits to meet the bioenergetic and biosynthetic demands of rapid cell proliferation and adaptation to the new microenvironment (7). In addition to cancer cells, evolving research has found that EMT can also give immune cells unique metabolic characteristics that affect their immunoregulatory function in response to cancer development (8). Thus, a better understanding of the interdependence between EMT and tumor metabolism could facilitate the discovery of effective predictive markers and new approaches to improve outcomes of BLCA.

In our study, we divided BLCA patients of the TCGA cohort into subgroups with different EMT status and metabolic characteristics. Based on the difference analysis between subgroups, we applied a variety of bioinformatics methods to establish an immune-related gene signature which can stably predict the prognosis and the response to chemotherapy and immunotherapy of patients. Our findings may help to optimize risk stratification of patients and provide a basis for studying the interplay between EMT and metabolic reprogramming.

## Materials and methods

### Data acquisition and preprocessing

RNA sequencing profile, mutation annotation format (MAF) file and corresponding clinical data of BLCA patients were downloaded from The Cancer Genome Atlas (https://portal.gdc.cancer.gov). Validation datasets GSE31684 and GSE32894 were obtained from the GEO data portal (https://www.ncbi.nlm.nih.gov/geo/). Validation dataset IMvigor210 were obtained from IMvigor210CoreBiologies, a fully documented R package (9). All the patients from the IMvigor210 cohort received at least one dose of ICI therapy. “ESTIMATE” R package was used to calculate immune/stromal scores for TCGA patients (10). Protein expression information for BLCA patients of TCGA cohort was obtained from the TCPA portal (https://www.tcpaportal.org/). Gene mutation information was obtained from the cBioPortal portal (http://www.cbioportal.org/). Immunohistochemical information was obtained from the HPA portal (https://www.proteinatlas.org/).

### EMT and metabolic signature analysis

The prognostic value of various EMT signatures in numerous studies was evaluated through the EMTome portal (http://www.emtome.org/) (11). Then we obtained the EMT signature containing 200 genes from the MSigDB portal (http://software.broadinstitute.org/gsea/msigdb). Seven metabolic gene signatures were obtained from a previous study (12). These gene sets are mostly independent of each other and represent the major metabolic processes, including amino acid metabolism, carbohydrate metabolism, integration of energy, lipid metabolism, nucleotide metabolism, tricarboxylic acid cycle (TCA) and vitamin & cofactor metabolism. The activity scores of the metabolic pathways for each sample of TCGA cohort were calculated by single-sample gene set enrichment analysis (ssGSEA) with the R package GSVA. The results of ssGSEA were standardized by linear normalization.

### Identification of molecular subtypes based on EMT and metabolism scores

According to the results of ssGSEA, the TCGA cohort was clustered into different subgroups by “Sparcl” R package. The differentially expressed gene (DEGs) between the two most significantly different clusters were analyzed with “Limma” R package. The adjust P < 0.05 and |log_2_(Fold Change) | > 1 were set as the cut‐off criteria to screen for DEGs. Then we downloaded a list of immune genes from the Immport portal (https://www.immport.org/) and intersected them with the DEGs to obtain immune-related genes that changed under different EMT and metabolic states (13). These genes were included in further analysis.

### Immune infiltration analysis, Gene Set Enrichment Analysis (GSEA) and function annotation

Comparison of 22 different types of immune cell fractions between subgroups were conducted by using “CIBERSORT” script in R (14). GSEA was performed by “ClusterProfiler” R package. The reference gene sets were downloaded from the MSigDB portal. Gene Ontology (GO) analysis and Kyoto Encyclopedia of Genes and Genomes (KEGG) pathway enrichment analysis were also performed by “ClusterProfiler” R package. Pathways with FDR (false discovery rate) < 0.25 and P < 0.05 were considered statistically enriched.

### Construction and validation of the prognostic gene signature

First, univariate Cox analysis of overall survival (OS) was performed to screen out the immune-related DEGs with prognostic value. Subsequently, we performed Lasso-penalized regression analysis with the “glmnet” R package to select the genes for constructing the predictive model. Finally, stepwise multivariate Cox regression analysis was applied to determine signature genes and their relative coefficient. The risk score (RS) for each patient was calculated as follows:


$$\text{RS}=\sum_{\text{i}}\text{Coefficient}\left(\text{mRNA}\right)\times \text{Expression}\left({\text{mRNA}}_{\text{i}}\right)$$

The training and validation cohorts were divided into high-risk and low-risk groups based on the median RS calculated separately. Kaplan-Meier survival analysis was conducted to assess the survival differences between risk groups. Time-dependent receiver operating characteristic curves (ROC) was implemented using the “survivalROC” R package to evaluate the sensitivity and specificity of the signature. Nomogram was made by “regplot” R package, and time-dependent AUC curves were drawn by “timeROC” R package.

### Assessment of drug response based on the signature

The response of the different risk groups to chemotherapy was validated by the TCGA cohort. The TIDE algorithm was used to evaluate the sensitivity of immunotherapy in BLCA patients in the TCGA cohort (15). Patients with TIDE values >0 were defined as non-responders (negative sensitivity), while TIDE values <0 were defined as responders (positive sensitivity). We also downloaded the immunophenoscore (IPS) from the TCIA portal (https://tcia.at) for the TCGA cohort to assess the sensitivity to immune checkpoint in different risk groups (16). Moreover, the independent cohort IMvigor210 was used to test the ability of the signature for predicting the immunotherapeutic response.

### Cell culture and transfection

The human bladder cancer cell lines T24 and UMUC3 were provided by the Cell Bank of the Chinese Academy of Sciences (Shanghai, China). The cell lines were cultured in RPMI 1640 medium supplemented with 10% foetal bovine serum (FBS; Gibco, Grand Island, NY, USA) at 37°C under 5% CO2 in a humidified incubator. For transient knockdown, the specific small interfering RNA (siRNA) for AHNAK and NFATC1 were purchased from GenePharma (Shanghai, China) and the sequences are shown in **Supplementary Table 1**. Cells were transfected with siRNA using Lipofectamine 3000 (Thermo Fisher Scientific) for 24h according to manufacturer’s instructions.

### Quantitative real-time PCR and western blotting

Total RNA was extracted using TRIzol (Takara) and cDNA of each group was synthesized with the PrimeScript™ RT reagent kit (Takara). qPCR was performed with the Roche LightCycler 480II real-time PCR detection system (Roche, Basel, Switzerland). Expression level of each gene was normalized to that of β-actin. The primers for qRT-PCR are listed in **Supplementary Table 2**.

Western blotting (WB) was performed as described previously (17). The antibodies used were as follows: anti-AHNAK antibody (sc-390743, Santa Cruz Biotechnology), anti-NFATC1 antibody (sc-7294, Santa Cruz Biotechnology), anti-E-Cadherin antibody (20874-1-AP, Proteintech), anti-Vimentin antibody (#5741, Cell Signaling Technology), anti-PFKFB3 antibody (ab181861, Abcam), anti-LDHA antibody (#3582, Cell Signaling Technology), anti-GLS antibody (ab156876, Abcam), anti-GLUD1 antibody (ab168352, Abcam), anti-PDL1 antibody (#13684S, Cell Signaling Technology), anti-α-actinin antibody (11313-2-AP, Proteintech) and anti-β-actin antibody (#4970, Cell Signaling Technology).

### Cell proliferation, migration and invasion assays

MTT (#88417, Sigma-Aldrich)assay was used to detect cell proliferation. Cells were transfected with siRNA for 24 hours and then inoculated in 96-well plates, each well containing 200 μl of complete growth medium, and cultured for the indicated times. Then the number of viable cells of different groups were measured by MTT methods.

Cell migration and invasion assay were performed by transwell chamber technology. In the migration assay, cells (5× 10^4^) were inoculated in the upper chamber (Corning) with serum-free medium after siRNA transfection and the lower chamber was filled with medium containing 10% FBS. After 24 hours, the cells on the membrane of transwell inserts were fixed with 4% paraformaldehyde and stained with 0.1% crystal violet. The invasion assay followed the same procedure as the migration assay, except that the membrane was coated with Matrigel (BD Biosciences).

### Statistical analysis

Student’s t-test or one-way analysis of variance was used to analyze differences between groups in variables with normal distribution. Wilcoxon rank-sum test or Kruskal-Wallis test was for groups without normal distribution. P value < 0.05 was regarded as statistically significant (* p<0.05, ** p<0.01, *** p<0.001, **** p<0.0001).

## Results

### Activation of the EMT pathway is a risky prognostic factor for bladder cancer

**Figure 1:**
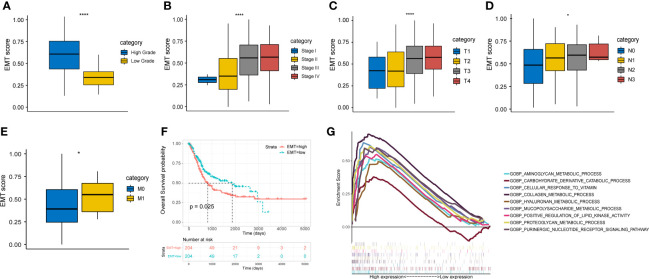
EMT score shows important clinical prognostic value and biological implication in TCGA cohort. **(A-E)** The correlation between EMT score and grade, clinical stage and AJCC TNM stage. **(F)** Comparison of overall survival between high and low EMT score groups. **(G)** Multiple metabolic regulatory pathways were significantly enriched in the high EMT score group. * p<0.05, **** p<0.0001.

All the gene sets used for ssGSEA analysis were presented in **Supplementary Table 3** and normalized ssGSEA results were presented in **Supplementary Table 4**. **Figures 1A-E** showed a significant correlation between EMT score and pathological grade, clinical stage, and TNM stage in the TCGA cohort. The higher EMT score corresponded to the higher degree of malignancy. In addition, we divided the patients of TCGA cohort into two groups based on the median EMT score. The prognosis for overall survival was significantly better in the low score group than in the high score group (**Figure 1F**). The GSEA results showed that multiple metabolic regulation-related pathways were significantly enriched in the high EMT score group, implying that tumor undergo EMT with concomitant metabolic reprogramming (**Figure 1G**). We also evaluated the prognostic value of various EMT gene sets in other studies and showed that EMT was indeed a risk prognostic factor (**Supplementary Figure 1**).

### Identification of molecular subgroups with different EMT activities and metabolic status

**Figure 2:**
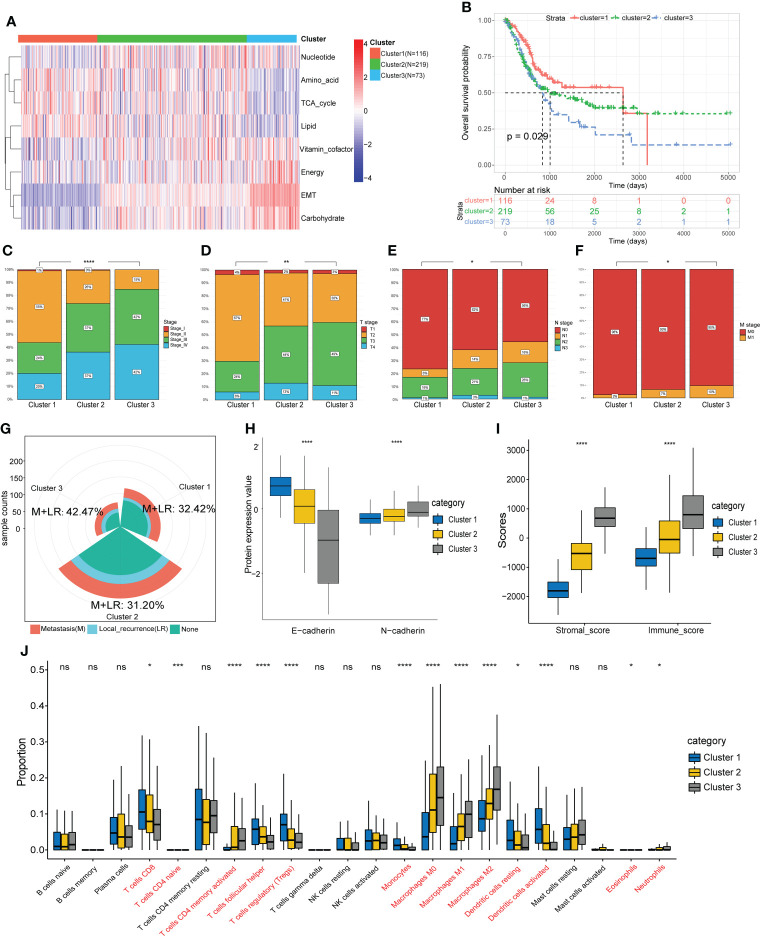
Identification of molecular subtypes based on EMT and metabolism scores. **(A)** The TCGA cohort was divided into three clusters based on cluster analysis of the ssGSEA scores. **(B)** Overall survival of the three clusters was compared by KM survival analysis. **(C-F)** Comparison of clinical characteristics between the three clusters. **(G)** Comparison of the proportion of metastasis and local recurrence after receiving chemotherapy. **(H)** Comparison of the expression levels of EMT marker proteins. **(I)** Comparison of the ESTIMATE scores. **(J)** Comparison of the infiltration of 22 leukocyte types among three clusters. *p<0.05, ** p<0.01, *** p<0.001, **** p<0.0001, ns: no significance.

Based on the EMT and metabolic scores derived from the ssGSEA algorithm, the total 408 patients in the TCGA cohort were clustered into three groups, where distinct states existed for cluster 1 (N=116) and cluster 3 (N=73). Cluster 3 had significantly higher scores in EMT, energy and carbohydrate than cluster 1, while scores in amino acid, TCA cycle and lipid were lower than cluster 1(**Figure 2A**). In addition, cluster 3 showed the worst prognosis (**Figure 2B**). Then we explored the association between the three subgroups and clinical characteristics. The results showed a stepwise increase in the proportion of high clinical stage and TNM stage from cluster 1 to cluster 3 (**Figures 2C-F**). Cluster 3 also had the highest rate of local recurrence and metastasis among patients who had received chemotherapy (**Figure 2G**). At the protein level, cluster 3 showed a stronger tendency toward EMT (**Figure 2H**). As assessed by the ESTIMATE algorithm, cluster 3 had a significantly higher immune score and stromal score than the other clusters (**Figure 2I**). Moreover, immune infiltration analysis showed a lower percentage of CD8^+^ T cells and a higher percentage of M2 macrophages in cluster 3, suggesting that the tumor microenvironment differed among clusters and cluster 3 expressed the lowest level of immunity (**Figure 2J**).

### Immune-related DEGs screening and biological function annotation

**Figure 3:**
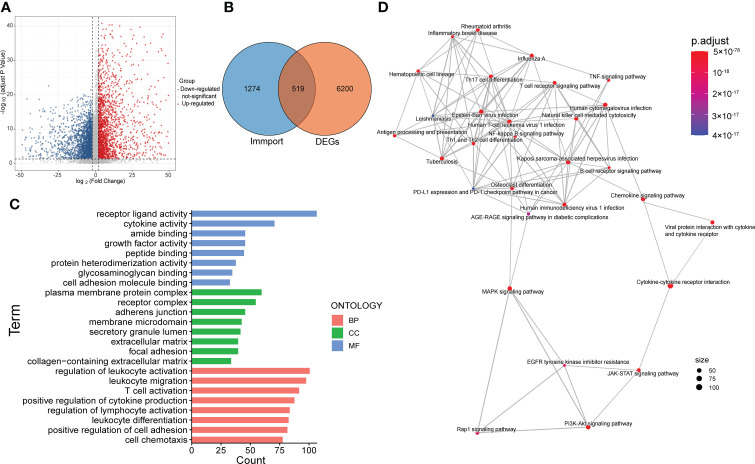
Screening and functional enrichment analysis of immune-related DEGs. **(A)** Volcano map showed the DEGs between cluster 3 and cluster 1. **(B)** Venn diagram displayed the amount of immune-related DEGs. **(C)** Top 8 items of molecular function (MF), cellular component (CC) and biological process (BP) of GO analysis. **(D)** The result of KEGG pathway analysis.

For further exploration, we performed differential gene analysis between cluster 3 and cluster 1 and obtained 6719 DEGs, which were subsequently intersected with the immune gene list from Immport to obtain a total of 519 immune-associated DEGs (**Figures 3A, **). Then we performed GO and KEGG analysis for the 519 genes. The results of molecular function (MF) and cellular component (CC) focused on pathways related to molecular interactions and cell adhesion, while the biological processes (BP) were mainly involved in the regulation of the immune system (**Figure 3C**). The results of KEGG analysis encompassed pathways associated with tumor malignancy regulation and immune modulation, such as MAPK signaling pathway, PI3K-Akt signaling pathway, T cell receptor signaling pathway and PDL1/PD1 checkpoint pathway (**Figure 3D**).

### Construction and validation of the immune signature associated with EMT and metabolic reprogramming

**Figure 4:**
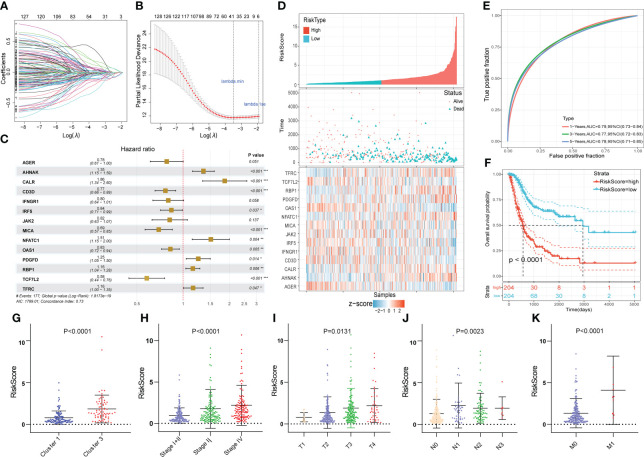
Construction of the novel immune-related gene signature based on EMT and metabolic status. **(A, B)** The LASSO regression was used to reduce the dimensionality of survival related genes after univariable Cox survival analysis. **(C)** Stepwise multivariate Cox regression analysis to construct the 14-gene prognostic signature. **(D)** The heatmap of the 14-gene expression and the distribution of patient survival status ranked by corresponding RS. **(E)** The time-dependent ROC curve for predicting 1-, 3-, and 5-year overall survival in TCGA cohort. **(F)** The KM survival analysis between high and low RS groups. **(G-K)** The correlation between RS and clusters, clinical stage and AJCC TNM stage. *p<0.05, **p<0.01, ***p<0.001.

The 519 immune-associated DEGs were included in the model construction process. First, univariate Cox survival analysis was applied to deal with these genes and 129 genes had significant prognostic value. Then 37 genes were retained after LASSO regression analysis and these genes were used for stepwise multivariate Cox regression analysis to construct the model (**Figures 4A**, **B**). Ultimately, 14 immune-related DEGs were selected for the construction of the novel prognostic gene signature (**Figure 4C**). Risk scores were calculated for each sample based on the expression levels and coefficients of these 14 genes as follows: RS = (-0.246 × AGER) + (0.303 × AHNAK) + (0.623 × CALR) + (-0.266 × CD3D) + (-0.222 × IFNGR1) + (-0.176 × IRF5) + (-0.201× JAK2) + (-0.366 × MICA) + (0.415 × NFATC1) + (-0.191 × OAS1) + (0.226 × PDGFD) + (0.146 × RBP1) + (-0.545 × TCF7L2) + (-0.150 × TFRC). The TCGA cohort was divided into high- and low- risk groups based on the median value of RS. The score distribution and survival status of all patients and the expression levels of 14 genes were shown in **Figure 4D**. The 1-, 3-, and 5-years area under curve (AUC) values of OS were 0.79, 0.77, and 0.78, respectively (**Figure 4E**). The prognosis of patients in the high RS group was significantly worse than that of the low RS group (**Figure 4F**). Moreover, RS were closely correlated with clinical characteristics (**Figures 4G-K**).

**Figure 5:**
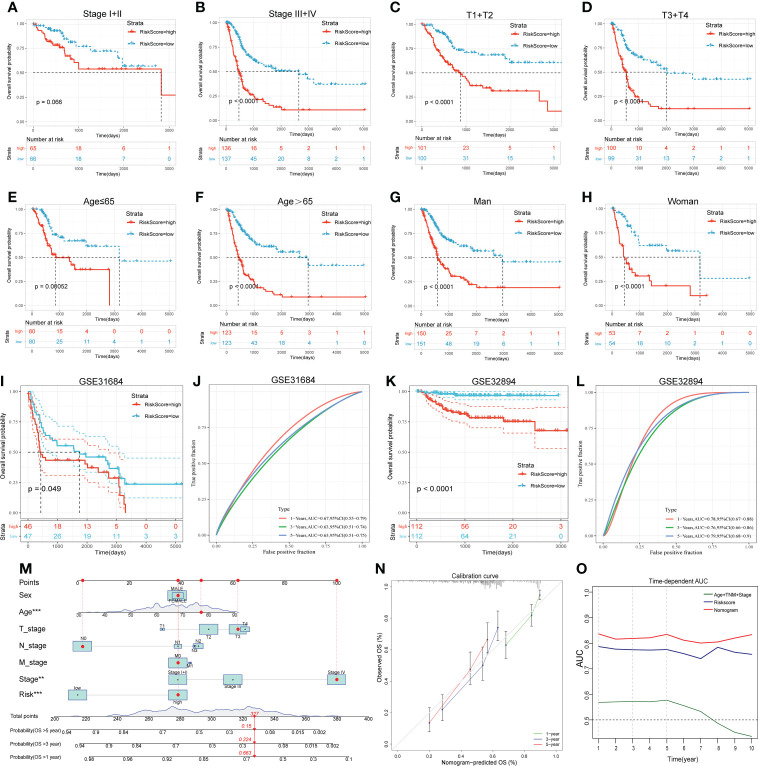
Subgroup analysis, external validation and nomogram proved the predictive value of the 14-gene signature. **(A-H)** The KM survival curves of OS between high and low RS groups in different subgroups. **(I-L)** The KM survival curves of OS between high and low RS groups and the time-dependent ROC curves of validation cohorts. I and J, GSE31684. K and L, GSE32894. **(M)** The nomogram created by integrating clinical information and Riskscore. **(N)** Calibration curve of the nomogram. **(O)** Time-dependent AUC curves of different prognostic models.

For subgroup validation, BLCA patients in the TCGA cohort were divided into different groups based on the following characteristics: age, gender, clinical stage, and AJCC T stage. Patients in the low RS group can obtain better survival benefits, suggesting that the signature had robust predictive ability in different subgroups (**Figures 5A-H**). We also validated the predictive efficacy of the signature with two other independent cohorts GSE31684 and GSE32894 (**Figures 5I**, **K**). The 1-, 3-, and 5-years AUC values of OS were 0.67, 0.63, 0.63 in GSE31684 and 0.78, 0.76, 0.79 in GSE32894 (**Figures 5J**, **L**). Moreover, we also integrated the clinical information of TCGA cohort with the riskscore to build a nomogram model for predicting the survival probability (**Figure 5M**). The red dots showed how to use this nomogram to calculate the survival probability for a patient. The calibration curve showed that the nomogram had a high prediction accuracy (**Figure 5N**). The nomogram had higher AUC value compared to riskscore alone, suggesting that our gene signature had better predictive potential when combined with clinical factors (**Figure 5O**).

### Comparison the immune infiltration, metabolic status and pathway enrichment between high- and low- risk groups

**Figure 6:**
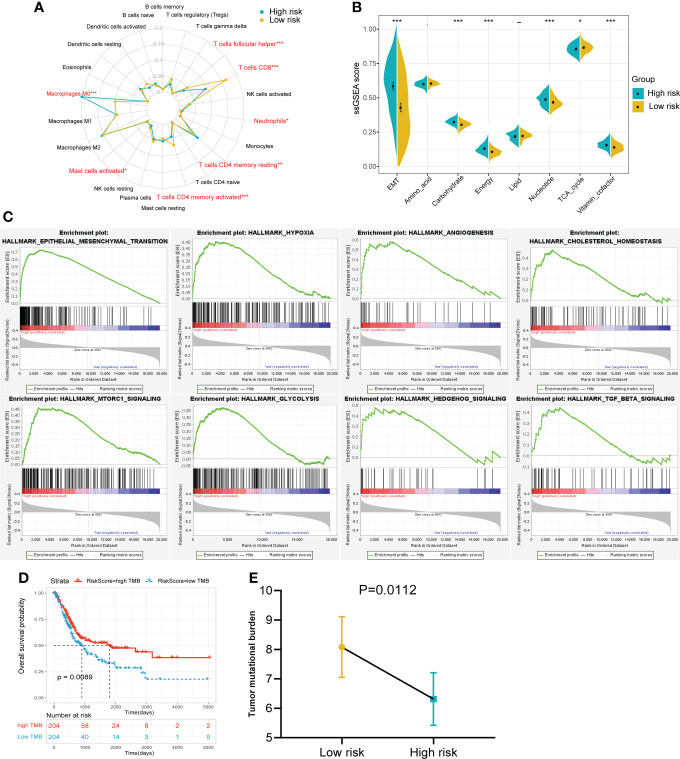
The immune infiltration, metabolic status and pathway enrichment between high and low risk groups in TCGA cohort. **(A)** Comparison of 22 types of immune cell infiltration. **(B)** Comparison of various metabolic scores of ssGSEA result. **(C)** Multiple malignant regulatory pathways were significantly enriched in the high risk group. **(D)** The KM survival curve of OS between high and low TMB groups. **(E)** Comparison of the TMB value between risk groups. *p<0.05, ***p<0.001.

To further explore the differences between the risk groups, a series of in-depth studies were conducted. Immune infiltration analysis showed a higher percentage of CD8^+^ T cells in the low-risk group (**Figure 6A**). The high-risk group was more inclined to undergo EMT, high carbohydrate and energy metabolism, which was very similar to cluster 3 (**Figure 6B**). The GSEA results also revealed that multiple cancer-related pathways were significantly enriched in the high-risk group, such as EMT, hypoxia, angiogenesis and glycolysis (**Figure 6C**). Tumor mutational burden (TMB), another biomarker that predicts the effect of immunotherapy, is also an important prognostic marker (**Figure 6D**). Patients in the low-risk group had a significantly higher TMB, implying that patients with low RS may be more likely to benefit from immunotherapy (**Figure 6E**). In addition, the interrelationships between the 14 genes in the signature and their relationships with metabolic scores were shown in **Supplementary Figure 2**.

### Prognostic value of the gene signature to chemotherapy and immunotherapy

**Figure 7:**
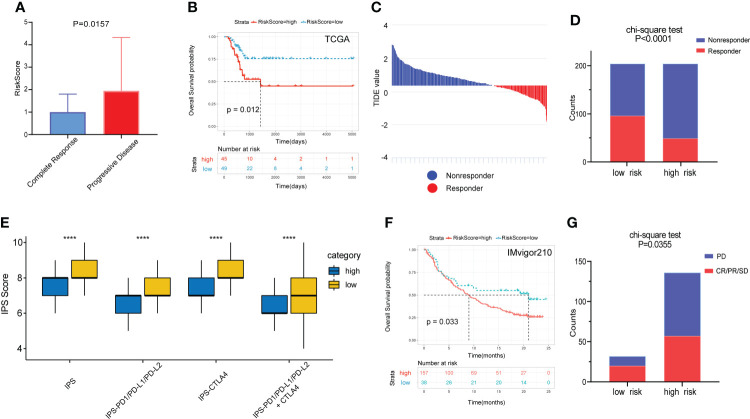
Prognostic value of the gene signature to chemotherapy and immunotherapy. **(A)** Comparison of RS among patients with complete response and progressive disease after chemotherapy. **(B)** The KM survival curve of OS between high and low RS groups in patients after chemotherapy. **(C)** Distribution of TIDE value in TCGA cohort. **(D)** Comparison of the proportion of responders and non-responders in different RS groups of TCGA cohort. **(E)** The association between IPS and RS groups. **(F)** The KM survival curve of OS between high and low RS groups in the IMvigor210 cohort. **(G)** Comparison of outcomes after receiving immunotherapy in different RS groups in the IMgivor210 cohort. ****p<0.0001.

Of the 94 patients in the TCGA cohort who had received chemotherapy, 44 were in complete response and 37 had progressive disease. The risk score of patients with complete response was significantly lower than that of patients with progressive disease. (**Figure 7A**). Among patients who had received chemotherapy, those with high RS had a significantly poorer OS benefit (**Figure 7B**). Then we predicted the response of patients in the TCGA cohort to immunotherapy by the TIDE algorithm (**Figure 7C**). The results showed a higher percentage of responders in the low-risk group (**Figure 7D**).

The IPS scores of patients in the low-risk group for multiple immune checkpoints were also significantly higher than those in the high-risk group, suggesting that the gene signature may be helpful to predict the response of patients to immunotherapy (**Figure 7E**). Therefore, we validated this value using patients with bladder cancer in the IMvigor210 immunotherapy cohort. Patients in the high-risk group had a significantly poorer prognosis and a much lower response rate to immunotherapy than those in the low-risk group (**Figures 7F, G**).

### Signature genes AHNAK and NFATC1 were closely related to EMT as well as metabolism in BLCA cell lines

**Figure 8:**
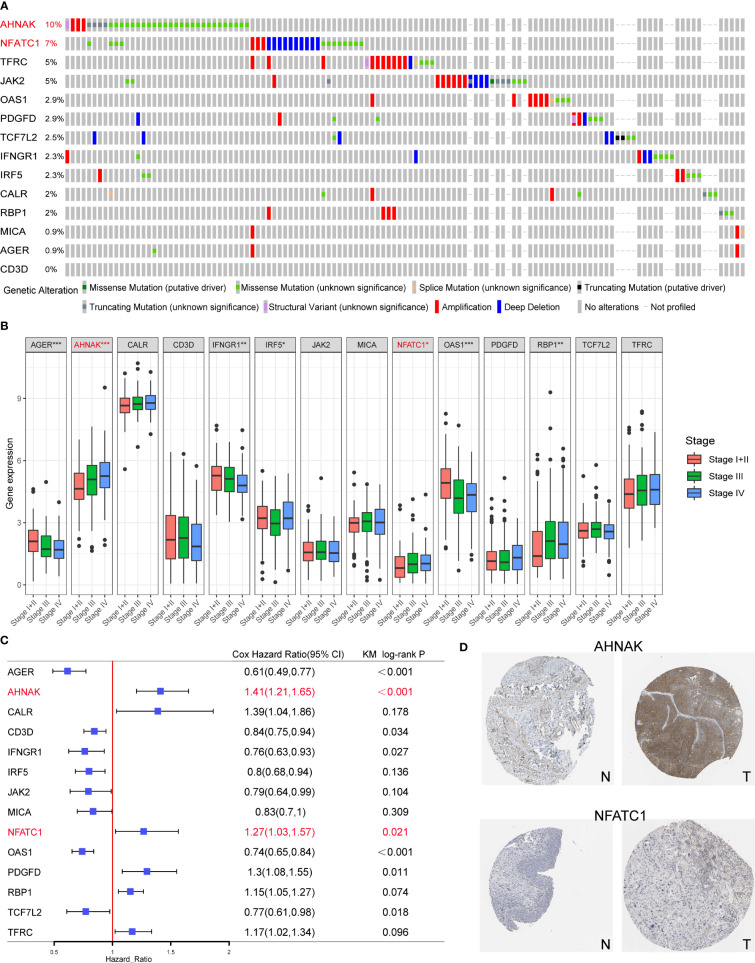
Screening of the key model genes for further experimental validation. **(A)** Mutation frequencies of the 14 genes in the TCGA cohort. **(B)** The correlation between the 14 genes and clinical stage of TCGA BLCA patients. **(C)** The results of univariate Cox and KM survival analyses for the 14 genes. **(D)** Expression levels of AHNAK and NFATC1 in cancerous and normal tissues. N, normal; T, tumor. *p<0.05, **p<0.01, ***p<0.001.

Among the 14 genes in the model, AHNAK and NFATC1 had the highest mutation frequencies and were closely associated with clinical stage (**Figures 8A**, **B**). Univariate Cox and KM survival analysis showed that they were risk prognostic factors (**Figure 8C**). In addition, both genes were expressed at higher levels in bladder cancer tissues than in normal tissues (**Figure 8D**). Therefore, we experimentally validated the functions related to EMT, glycolysis, glutamine metabolism and immune checkpoint regulation of AHNAK and NFATC1 in bladder cancer.

**Figure 9:**
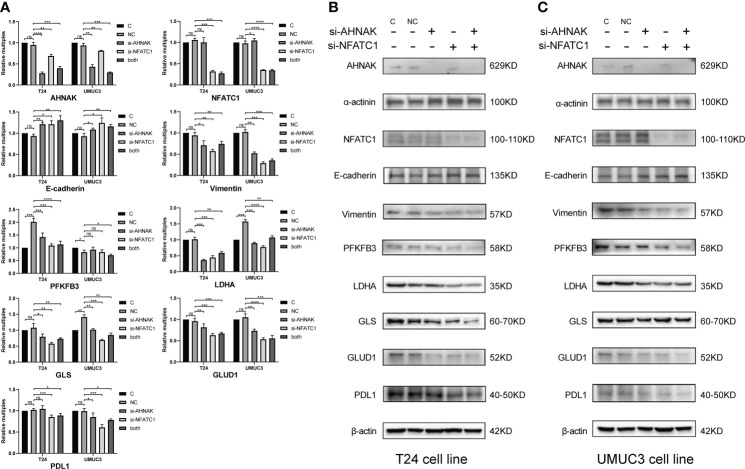
qPCR **(A)** and WB **(B, C)** were used to detect the effects of AHNAK and NFATC1 knockdown with siRNAs on key glycolysis enzymes, amino acid metabolism enzymes, EMT and PD-L1 immune checkpoints of two bladder cancer cell lines T24 and UMUC3. Glycolysis enzymes involve PFKFB3 and LDHA. Glutamine metabolic enzymes involve GLS and GLUD1. EMT involves E-cadherin and vimentin. β-actin and α-actin were used as internal references. *p<0.05, **p<0.01, ***p<0.001, ****p<0.0001. ns, no significance.

As shown in **Figure 9**, we performed qRT-PCR and WB experiments in both T24 and UMUC3 cell lines. When AHNAK and NFATC1 were knocked down, the EMT marker E-cadherin was increased while vimentin was downregulated. The key enzymes of glycolysis, PFKFB3 and LDHA, and the key enzymes of glutamine metabolism, GLS and GLUD1, were significantly downregulated in at least one cell line. The level of PDL1 was also significantly downregulated in both cell lines. These results suggested a close association of AHNAK and NFATC1 with EMT genesis, metabolic reprogramming, and immune escape in bladder cancer.

### AHNAK and NFATC1 can promote the migration, invasion and proliferation of BLCA cell lines

**Figure 10:**
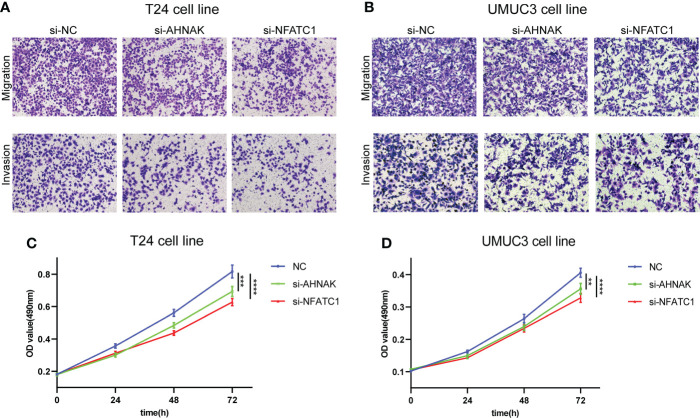
AHNAK and NFATC1 increases cell migration, invasion and proliferation in BLCA. **(A, B)** Transwell migration and invasion assays of T24 and UMUC3 cells transfected with siRNAs against AHNAK and NFATC1 (100× magnification). **(C, D)** MTT assay of T24 and UMUC3 cells transfected with siRNAs against AHNAK and NFATC1. **p<0.01, ***p<0.001, ****p<0.0001.

To investigate the effects of AHNAK and NFATC1 on the biological functions of bladder cancer cells, we performed transwell and MTT assays. The ability of cancer cells to migrate and invade was significantly inhibited after AHNAK or NFATC1 was knocked down (**Figures 10A**, **B**). Besides, the proliferation rate of cancer cells was significantly decreased after AHNAK and NFATC1 knockdown (**Figures 10C**, **D**). Based on these results, we believed that these two genes could influence the biological behavior of bladder cancer cells and were of significant research value.

## Discussion

EMT is a fundamental biological process involved in development. Through the regulation of EMT, systems, organs and tissues are formed and their function, growth and regeneration are maintained (18). However, dysregulated EMT can lead to disease, including dysplasia, fibrosis, and tumorigenesis (19). EMT-transformed bladder cancer cells have been reported to possess stem cell properties. Induction of EMT not only promotes the proliferation of tumor cells from their primary sites, but also enhances the self-renewal ability of tumor cells and confers them with greater migration ability (5). It is reasonable to expect that altering the motor phenotype will require alterations in cellular bioenergetics and thus metabolism. Indeed, the link between EMT and metabolism is reciprocal, and the extensive regulatory crosstalk between the two has been intensively studied (20).

Metabolic reprogramming of tumors refers to the construction of a completely new metabolic network under the aberrant expression of oncogenes, which redefines the flow of nutrients and energy in the metabolic network during tumorigenesis (M. 21). Metabolic reprogramming is an important pathway that mediates EMT while itself being strictly regulated by EMT-related factors. Cancer cells meet their specific energy needs by regulating their metabolism and synthesizing biomolecules including proteins, lipids and nucleic acids (22). Metabolic adaptation in cancer cells involves many key metabolic pathways, most notably glycolysis, TCA cycle, lipid and amino acid metabolism, which can directly regulate the dynamics of EMT and are closely associated with cancer cell survival, invasion and metastasis (6).

Metabolic reprogramming maintains the Warburg effect of tumors and induces EMT by enhancing glycolysis and blocking the TCA cycle (23). Malignant tumor cells are able to promote the increase of glucose transport proteins thereby enhancing aerobic glycolysis and maintaining their metastatic potential. Among these transporter proteins, GLUT1 and GLUT3, induced by hypoxia-inducible factor 1α (HIF-1α), have been shown to potentiate glycolysis and cancer progression (24). Mitochondrial dysfunction often exists in tumor cells, resulting in lactic acid accumulation, which promotes the formation of acidic microenvironment and the progress of EMT. At the same time, EMT-induced stimulants also accelerate microenvironment acidification by triggering metabolic changes ( 25). It was also found that tumor cells have significant dysregulation of lipid metabolism, including high lipogenesis and low lipolytic capacity, increased membrane lipid synthesis, and upregulation of bioactive lipid, which induces the EMT process (23, 26). In addition, the microenvironment reshaped by metabolic reprogramming can affect the function of immune cells, allowing cancer cells to escape immune surveillance and leading to resistance to immunotherapy. The acidic tumor microenvironment is deficient in glucose, tryptophan and arginine, while the concentration of immunosuppressive molecules such as lactate and kynurenine are increased (27). Excess lactate promotes macrophage polarization to an inhibitory M2 phenotype and inhibits monocyte migration and differentiation into dendritic cells, thereby inhibiting antigen presentation and subsequent T cell activation (28). Lactate and kynurenine can also directly inhibit T cell-mediated immune responses (29).

In the present study, we combined the advantages of high-throughput sequencing to quantify EMT and various metabolic pathways using transcriptomic data and classified the TCGA bladder cancer cohort into three clusters. Among them, cluster 3 showed a distinctively different EMT and metabolic status compared to cluster 1. Patients in cluster 3 had the highest degree of malignancy and the worst prognosis. And other results were consistent with the description above, with a significant EMT trend and high energy/carbohydrate metabolism in cluster 3 and a significant inhibition of TCA cycle and lipid metabolism. Multiple cancer-related pathways were active in cluster 3, with a significantly lower proportion of CD8^+^ T cells. Then, we established a novel predictive signature with 14 genes by analyzing the differences between cluster 3 and 1, combining with the immune database and using multiple convergence methods. The model-calculated RS was strongly associated with clinical features, EMT and metabolic scores, TMB and immune infiltration. The model has good predictive power for overall survival in subgroup analysis as well as in external cohorts. More importantly, the predictive model can help to identify whether patients are able to respond to chemotherapy or immunotherapy. All these suggests that our risk score is a reliable predictor.

With the advent of bioinformatics era, there have been many prediction models and subtype classification methods based on various sequencing data (30–32). This study was the first combined analysis of EMT, metabolism and immunity in bladder cancer, and the results showed the robustness of the predictive model. Many of the genes in the model have been reported in previous studies, among which AHNAK and NFATC1 are the focus of our attention. The nuclear protein AHNAK, also known as desmoyokin, is a large complex scaffold protein with a tripartite nature and multiple domains. AHNAK has been reported to be involved in a variety of biological processes, including cell signaling and contacts, regulation of calcium channels, membrane repair and tumor metastasis (33). Shankar et al. reported that AHNAK was an important regulator of pseudopodia formation in metastatic cells, and knocking down AHNAK could reduce actin cytoskeletal dynamics and inhibit migration and EMT trends (34). In addition, AHNAK plays an important role in the regulation of glucose and lipid homeostasis, antigen presentation, and T cell activation (35, 36). NFATC1 is a transcription factor activated by the T cell receptor and Ca^2+^ signaling pathway that affects T cell activation and effector function (37). Oikawa et al. found that induced expression of NFATC1 downregulated E-cadherin expression and increased invasive activity in tumor xenografts *in vivo* (38). Liu et al. also found that inhibition of NFATC1 suppressed the proliferation, Warburg effect, migration and invasion of prostate cancer cells by down-regulating the expression of c-Myc and PKM2 (39).

Some limitations remain in this study. First, the predictive model in this study was obtained through retrospective analysis of public database, and its clinical validity remains to be verified in larger prospective trials. Second, our study only included some cell experiments, and more detailed *in vivo* and *in vitro* experiments are needed to explore the functions of these genes.

In conclusion, we constructed a novel gene signature related to EMT, metabolic reprogramming, and immunity that is effective in predicting the prognosis of bladder cancer patients and whether patients are able to respond to chemotherapy or immunotherapy. Our results provide a reference for the study of the interaction between EMT and metabolic reprogramming, and for the targeting of key metabolic molecules in the treatment of tumor metastasis.

## Data Availability Statement

The original contributions presented in the study are included in the article/**Supplementary Material**. Further inquiries can be directed to the corresponding authors.

## Ethics Statement

Any repository data used in this study are open access and do not require any permissions. Ethics approval and consent to participate are not applicable.

## Author Contributions

ZZ and HN contributed to conception and design. PL and MW contributed to collection and assembly of data. ZZ and WJ contributed to data analysis and interpretation. ZZ and YY contributed to manuscript writing. YL and HN contributed to the manuscript revision and finalization. All authors contributed to the article and approved the submitted version.

## Funding

This work was supported by the National Natural Science Foundation of China (82071750, 81972378, 81772713), Taishan Scholar Program of Shandong Province (tsqn20161077), Major Science and Technology Innovation Project of Shandong Province (2019JZZY021002), Shinan District Science and Technology Program of Qingdao (2022-2-002-YY), Scientific Development Fund of Dongying City (DJ2021032).

## Conflict of Interest

The authors declare that the research was conducted in the absence of any commercial or financial relationships that could be construed as a potential conflict of interest.

## Publisher’s Note

All claims expressed in this article are solely those of the authors and do not necessarily represent those of their affiliated organizations, or those of the publisher, the editors and the reviewers. Any product that may be evaluated in this article, or claim that may be made by its manufacturer, is not guaranteed or endorsed by the publisher.

## Supplementary material

Supplementary Figure 1 The prognostic value of various EMT signatures from numerous studies. The TCGA cohort is the validation cohort.

Supplementary Figure 2 Correlation between the 14 signature genes and their correlation with metabolic scores.

## Abbreviations

EMT, epithelial-mesenchymal transition; BLCA, bladder cancer; DEGs, differentially expressed genes; TCGA, the cancer genome atlas; MIBC, muscle invasive bladder cancer; NMIBC, non-muscle invasive bladder cancer; ssGSEA, single-sample gene set enrichment analysis; TMB, tumor mutation burden.
