# Supplementary figures and images for: Identification and validation of an immune signature associated with EMT and metabolic reprogramming for predicting prognosis and drug response in bladder cancer
Source: Front Immunol. 2022 Jul 25;13:954616. doi: 10.3389/fimmu.2022.954616 (PMC9359097; doi:10.3389/fimmu.2022.954616)

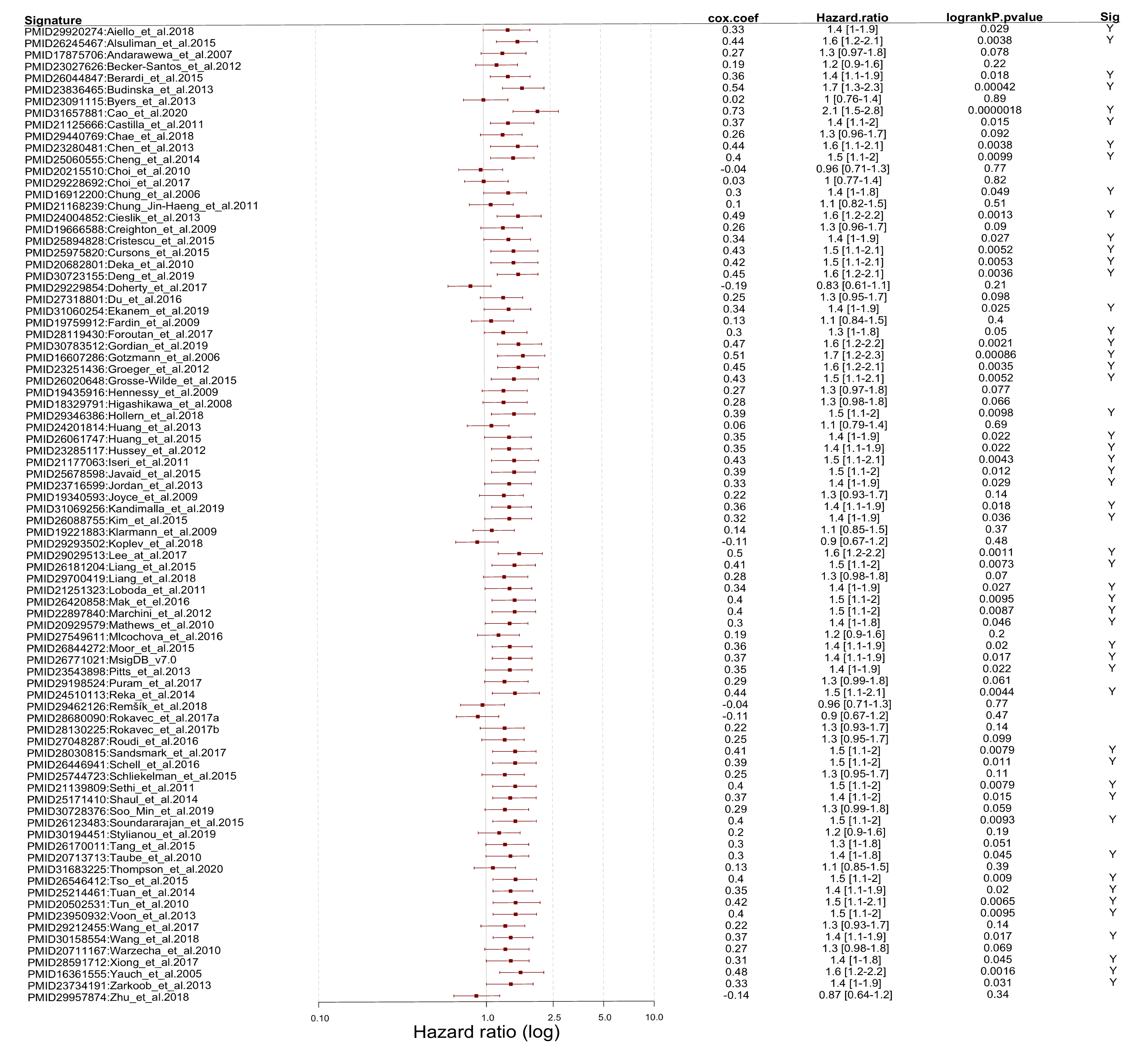

Supplement: Supplementary Figure 1 — The prognostic value of various EMT signatures from numerous studies. The TCGA cohort is the validation cohort. [file Image_1.tif]

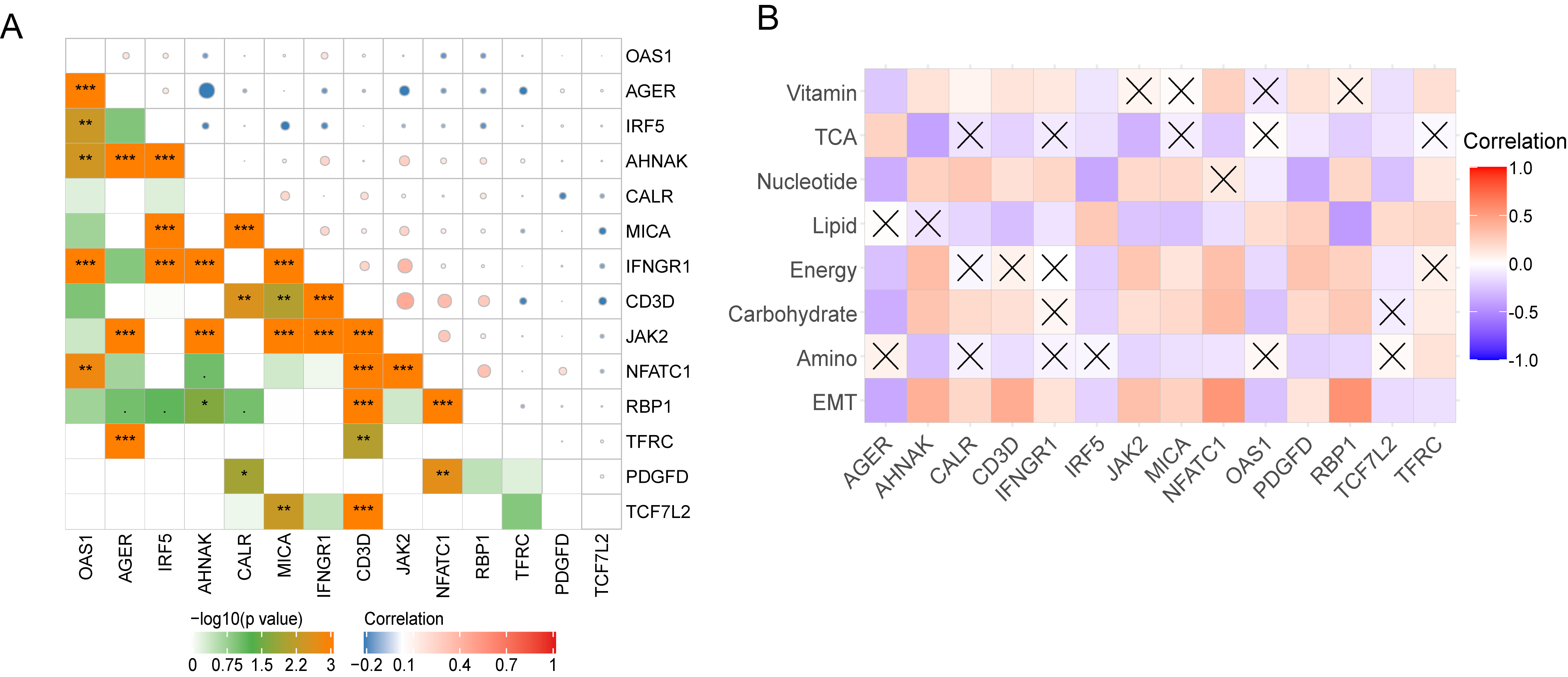

Supplement: Supplementary Figure 2 — Correlation between the 14 signature genes and their correlation with metabolic scores. [file Image_2.tif]
