# Supplementary material for: Identification and validation of an immune signature associated with EMT and metabolic reprogramming for predicting prognosis and drug response in bladder cancer
Source: Front Immunol. 2022 Jul 25;13:954616. doi: 10.3389/fimmu.2022.954616 (PMC9359097; doi:10.3389/fimmu.2022.954616)
Supplement: Supplementary file 3 [file Table_1.docx]

Supplementary Table 1. Small interfering RNA

| Gene | Primer | Sequence |
| --- | --- | --- |
| AHNAK | Sense | GGUUGAAAGUGAGAUUAAAGU |
|  | Antisense | UUUAAUCUCACUUUCAACCUU |
| NFATC1 | Sense | GGGACCUGUGCAAGCCGAAUUCUCU |
|  | Antisense | AGAGAAUUCGGCUUGCACAGGUCCC |
