# Supplementary material for: Identification and validation of an immune signature associated with EMT and metabolic reprogramming for predicting prognosis and drug response in bladder cancer
Source: Front Immunol. 2022 Jul 25;13:954616. doi: 10.3389/fimmu.2022.954616 (PMC9359097; doi:10.3389/fimmu.2022.954616)
Supplement: Supplementary file 4 [file Table_2.docx]

Supplementary Table 2. All primers for qRT-PCR

| Gene | Primer | Sequence (5' -> 3') |
| --- | --- | --- |
| AHNAK | Forward | AACTCAAGGGTCCAAAGTTCAAG |
|  | Reverse | GAGAGACATCCACATCACCTTTC |
| NFATC1 | Forward | TGTGCCGGAATCCTGAAACTC |
|  | Reverse | GAGCATTCGATGGGGTTGGAG |
| E-cadherin | Forward | TGCCCAGAAAATGAAAAAGG |
|  | Reverse | GTGTATGTGGCAATGCGTTC |
| Vimentin | Forward | TGCCCTTAAAGGAACCAATG |
|  | Reverse | TCCAGCAGCTTCCTGTAGGT |
| PFKFB3 | Forward | CTTGTCGCTGATCAAGGTGA |
|  | Reverse | TTCTGCTCCTCCACGAACTT |
| LDHA | Forward | TGGGAGTTCACCCATTAAGC |
|  | Reverse | AGCACTCTCAACCACCTGCT |
| GLS | Forward | GCTGTGCTCCATTGAAGTGA |
|  | Reverse | GCAAACTGCCCTGAGAAGTC |
| GLUD1 | Forward | GAATCCATGGACGCATCTCT |
|  | Reverse | TCCCATCAGACTCACCAACA |
| PDL1 | Forward | TATGGTGGTGCCGACTACAA |
|  | Reverse | TGCTTGTCCAGATGACTTCG |
| β-actin | Forward | GGCATCGTCACCAACTGGGAC |
|  | Reverse | CGATTTCCCGCTCGGCCGTGG |
